# Relaxation Dynamics of Large and Anisotropic Molecules in a Weakly Polar Matrix

**DOI:** 10.1021/acs.jpcb.6c03410

**Published:** 2026-10-08

**Authors:** Alfred Błażytko, Marzena Rams-Baron, Żaneta Wojnarowska, Marian Paluch

**Affiliations:** August Chełkowski Institute of Physics, 49568 University of Silesia, 75 Pulku Piechoty 1, Chorzów 41-500, Poland

## Abstract

The dynamics of large and anisotropic molecules embedded in glass-forming liquids present complex challenges due to the interplay between molecular shape, host–guest interactions, and macroscopic viscosity. In this work, we investigate fluorophenylene isomers (*ortho*-F and *para*-F) dissolved in the *ortho*-terphenyl (OTP) matrix, combining broadband dielectric spectroscopy, differential scanning calorimetry, and rheology to disentangle host and probe dynamics. While calorimetry reveals a single glass transition dominated by OTP, dielectric measurements resolve two distinct α-relaxation processes: a fast process corresponding to OTP structural relaxation and a slower process associated with probe relaxation, i.e., reorientation of large and anisotropic molecules in a weakly polar matrix. Furthermore, the α-relaxation peak of the guest molecules becomes noticeably narrower upon dilution in OTP. We attribute this behavior to the dynamic homogenization of the local environment arising from the large size mismatch between the guest and the host molecules. Mechanical relaxation times derived from viscosity match the fast dielectric α-process, indicating that OTP exclusively governs the viscous response. Interestingly, the *para*-F/OTP mixture shows ideal Debye–Stokes–Einstein (DSE) coupling, whereas *ortho*-F/OTP exhibits systematic decoupling, reflecting the importance of probe dipole orientation and anisotropy.

## Graphical abstract

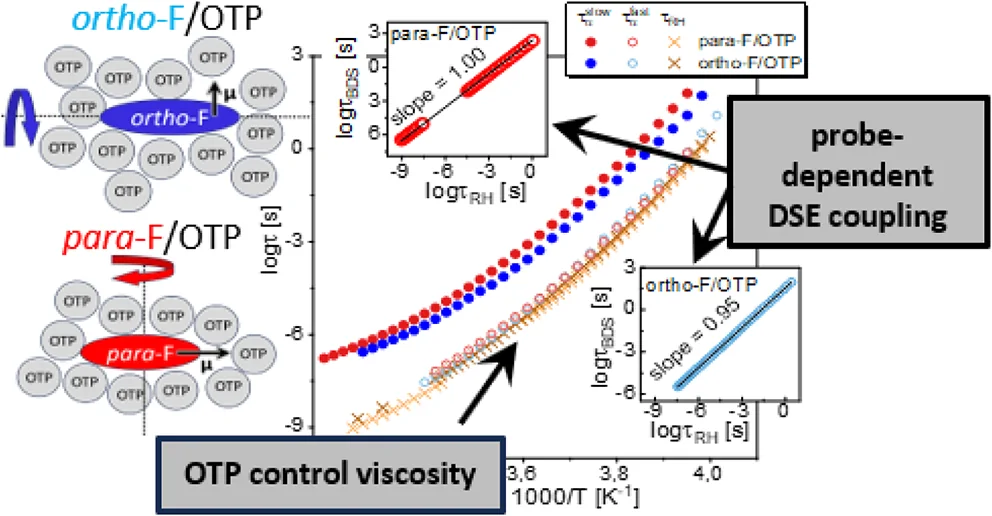

## Introduction

Understanding the dynamics of molecules in glass-forming liquids is a fundamental challenge in condensed matter physics, with implications for soft materials, pharmaceuticals, and functional nanocomposites. Molecular shape, size, and interactions dictate how individual molecules reorient, how structural relaxation occurs, and how macroscopic properties such as viscosity emerge.^1^ While much is known for small, nearly spherical molecules, systems containing large and highly anisotropic molecules display complex and often unexpected behavior, including decoupling between rotational dynamics and viscosity.

In our previous study, we demonstrated that in neat systems of large anisotropic molecules, the Debye–Stokes–Einstein (DSE) relation can either hold or break down depending on which molecular axis is probed, reflecting the dual nature of molecular relaxation.^2^ In the form adopted there, the DSE relation expresses the proportionality between the orientational relaxation time, τ, and viscosity, η, as $\tau \approx \frac{\eta }{T}$ which implies that molecular reorientation is governed by the same structural process that controls macroscopic viscous flow. Accordingly, if rotational and structural dynamics remain fully coupled, the orientational relaxation time should scale linearly with viscosity (or with the mechanical relaxation time derived from it).^3^ Our previous findings highlighted that anisotropic molecules can exhibit axis-dependent relaxation dynamics, with short- and long-axis reorientations corresponding differently to the surrounding viscous medium. In such systems, the effective hydrodynamic coupling may depend on the specific relaxation mode being probed, leading to either compliance with or deviations from the classical DSE prediction.^2^

Extending this approach, the present study focuses on mixtures of large fluorophenylene isomers (*ortho*-F and *para*-F) dissolved in a more weakly polar matrix, *ortho*-terphenyl (OTP). The size of OTP plays a critical role: by dynamically homogenizing the environment of the anisotropic probes, OTP reduces intermolecular interactions and modulates local molecular mobility. This allows us to probe how host size and probe anisotropy jointly influence relaxation dynamics, providing insights unattainable in neat systems.

Using a combination of broadband dielectric spectroscopy, differential scanning calorimetry, and rheological measurements, we resolve fast α-relaxation processes associated with the OTP matrix and slower α-relaxation processes corresponding to the sizable molecules. Remarkably, dilution in the OTP host leads to a pronounced narrowing of the dielectric α-relaxation spectra of the sizable molecules, reflected in increased β_KWW_ values. Furthermore, we observe distinct DSE behavior: the *para*-F/OTP mixture exhibits ideal coupling between probe relaxation and host viscosity, while the *ortho*-F/OTP mixture shows systematic decoupling across the supercooled temperature range.

This study extends the understanding of molecular dynamics from single-component systems to probe-host mixtures, emphasizing the critical influence of host molecular size, probe anisotropy and polarity of both components. The results demonstrate that a small and weakly polar host, such as OTP, can dynamically homogenize the environment, attenuate intermolecular interactions, and selectively modulate anisotropic molecular relaxation. These findings have broader implications for designing advanced systems where controlling the interplay between host and guest dynamics is essential.

## Materials and Methods

### Materials

In our studies we used two structural isomers *ortho*-F (N-{7-[(2-fluorophenyl)­ethynyl]-9,9-dibutyl-9H-fluoren-2-yl}-N,N-diphenylamine) and *para*-F (N-{7-[(4-fluorophenyl)­ethynyl]-9,9-dibutyl-9H-fluoren-2-yl}-N,N-diphenylamine), which were synthesized by TriMen Chemicals (Łódź, Poland) with a declared purity >97%. The molecular mass of this compounds is M = 563.75 g/mol. We also used *ortho*-terphenyl (M = 230.3 g/mol) from Sigma-Aldrich with a purity greater than 99% (see all molecular structures in Figure 1). Binary mixtures were prepared at a molar ratio of 1:10 (sizable molecule:OTP) by mixing the crystalline powders in a mortar until a homogeneous blend was obtained. In addition to the 1:10 mixtures used for the main BDS, DSC, and rheological analysis, additional *ortho*-F/OTP mixtures with molar ratios of 1:5, 1:1, and 5:1 were prepared to examine the influence of composition on the separation of the structural α-relaxation processes. All mixtures were prepared following the same procedure.

**Figure 1:**
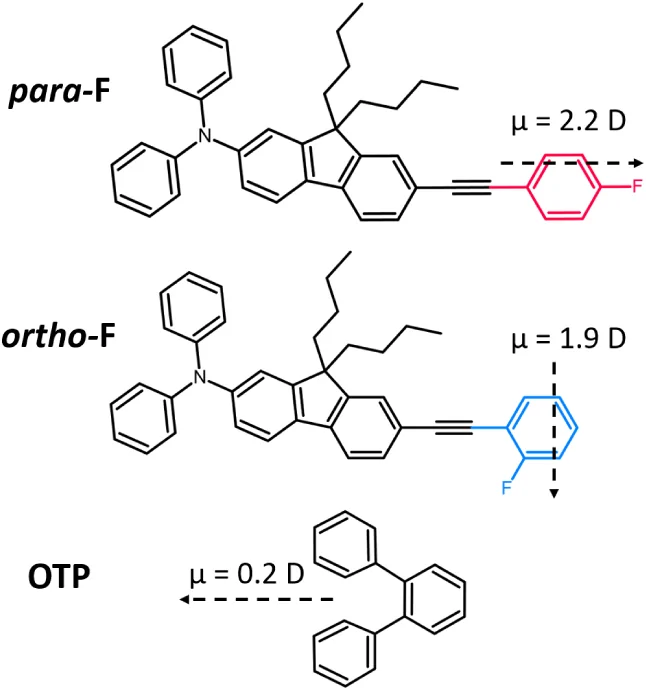
Structures of the investigated compounds. The dashed arrow symbolically indicates the dipole moment vector for each molecule.

### Differential Scanning Calorimetry

The thermal properties of the investigated materials were measured using a Mettler-Toledo differential scanning calorimeter equipped with a ceramic HSS8 sensor (120 thermocouples) and a liquid nitrogen cooling system. Enthalpy and temperature calibration were done by using zinc and indium standards. Samples (placed in 40 μL aluminum vessels) were measured in the crystalline and amorphous states with a heating rate of 10 K/min. The melting temperature was determined from the onset of the process, while the glass transition temperature was taken from the midpoint of the heat capacity step change. The tested temperature range was 240–373 K for OTP, 240–433 K for *ortho*-F and *para*-F, 240–423 K for *ortho*-F/OTP, and 240–433 K for *para*-F/OTP.

### Broadband Dielectric Spectroscopy

Complex electric permittivity ε*­(*f*) = *ε*′(*f*) – *iε*″(*f*) was measured using a Novocontrol GMBH Alpha spectrometer combined with Novocool Cryosystem, which was responsible for stabilizing the temperature to within 0.2 K. Samples were analyzed in the default frequency range of 10^–1^–10^6^ Hz; however, in temperatures close to the glass transition range, it was extended to 10^–3^ Hz. The investigated materials have been placed between parallel brass capacitor plates with a 40 mm diameter and a 0.05 mm gap provided by quartz fibers. Samples were melted on the bottom capacitor plate, dried under vacuum, and then covered with the top plate. Such a prepared capacitor was immediately placed in the spectrometer. The temperature range at which the dielectric data were analyzed was: for OTP 243–277 K, for *ortho*-F/OTP 251–303 K, and for *para*-F/OTP 253–313 K, with the step ΔT = 2 K. All ε″(f) spectra presented here have been shown without DC conductivity.

### Shear Rheology

Viscosity measurements were performed using an ARES G2 Rheometer and an aluminum parallel plate geometry of 8 mm diameter. Rheological testing was conducted over a frequency range of 0.1 to 100 rad·s^–1^ (10 points per decade) at a constant strain of 0.01% near the transition. The strain amplitude was increased by an order of magnitude every 10 K. The measurements were performed following two protocols: (i) on heating from the glassy state and (ii) on cooling of the molten sample. The relative combined uncertainty of the viscosity data, accounting for calibration, temperature and pressure control, and sample purity, was less than 7%.

## Results and Discussion

### Thermal Analysis

First, we characterized the thermal properties of the investigated materials using DSC. Figure 2a shows thermograms obtained during the heating of crystalline substances. All of the curves show endothermic processes that reflect melting. For neat OTP, the melting point is *T*_m_ = 329 K; for *ortho*-F, *T*_m_ = 408 K; and for *para*-F, two melting processes are observed at *T*_m_ = 387 K and *T*_m_ = 420 K, indicating the existence of two polymorphic forms of the compound. For *ortho*-F/OTP and *para*-F/OTP mixtures, the melting points are *T*_m_ = 324 K and *T*_m_ = 320 K, respectively. The apparent broadening of the melting process in the thermograms of the mixtures is typical for binary compositions, in which once one component has melted, the other begins to melt shortly thereafter. It is also worth noting that the mixtures have lower melting points than the pure substances, indicating the formation of a eutectic mixture.^4^ Figure 2b shows thermograms obtained during the heating of vitrified samples. Each thermogram shows a step change in heat capacity, which indicates a glass transition. For the neat substances OTP, *ortho*-F, and *para*-F, the glass transition temperatures are *T*_g_ = 249 K, *T*_g_ = 314 K, and *T*_g_ = 321 K, respectively. In the case of OTP, additionally, crystallization was observed at *T*_c_ = 290 K, followed by melting at *T*_m_ = 329 K. The results obtained for OTP are consistent with the literature data.^5^ Mixtures of *ortho*-F/OTP and *para*-F/OTP have a single glass transition at *T*_g_ = 252 K and *T*_g_ = 253 K, respectively.

**Figure 2:**
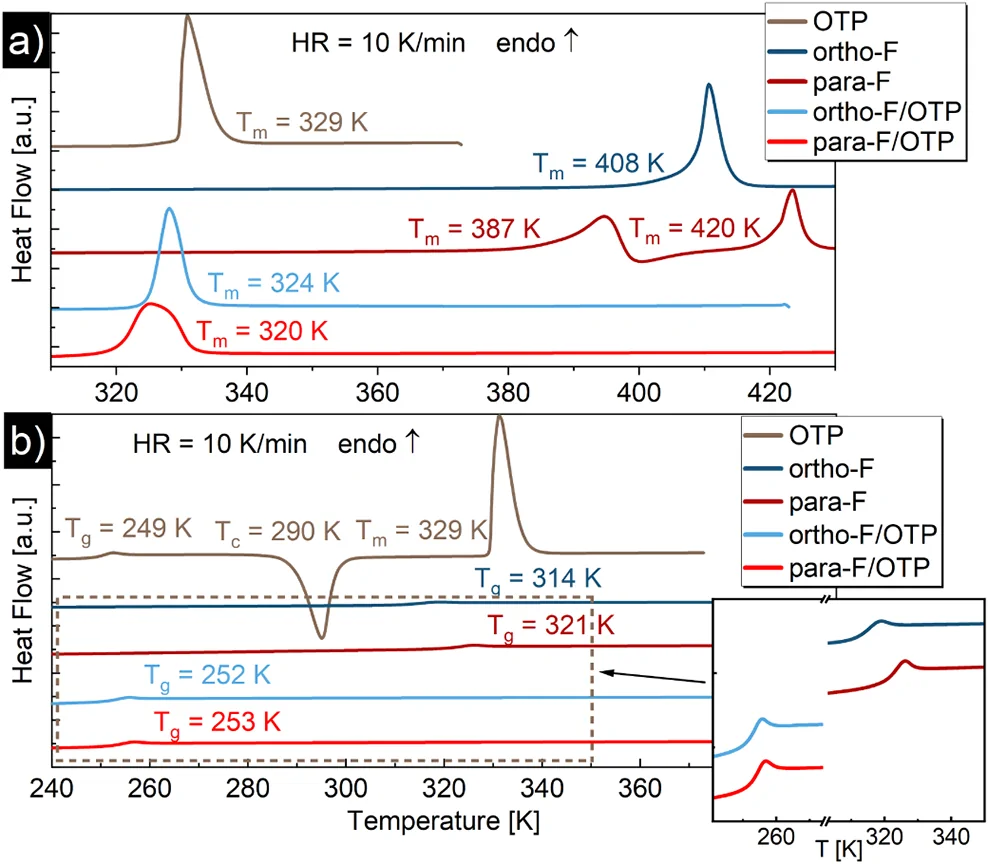
Thermograms of the investigated materials generated during heating of the (a) crystalline and (b) vitrified samples. The inset shows a close-up in the *T*_g_ region.

Typically, well-miscible binary mixtures exhibit a single glass transition (e.g., pharmaceuticals,^6–8^ polymers^9–11^ or H-bonded liquids).^12,13^ However, an increasing number of reports describe systems that, despite being miscible, exhibit two distinct glass transitions.^14–17^ For example, Körber et al. noted in their work that, in a mixture of large m-TPPS and relatively small TPP, two glass transitions were observed at all studied concentrations.^16^ In that case, the glass transitions of the individual components remain well separated due to an extreme mismatch in molecular size and dynamics. Because the components of the sizable-molecule/OTP mixture also differ significantly in size, a similar effect was expected in our systems as well. However, only a single calorimetric glass transition is observed for both *ortho*-F/OTP and *para*-F/OTP mixtures. The *T*_g_ values of the mixtures are only slightly shifted relative to neat OTP, indicating that the host matrix dominates the vitrification process. In this case, the substantially higher concentration of OTP gives rise to a dominant calorimetric signal that effectively masks the much weaker contribution from the sizable molecules. This is further supported by dielectric measurements, as discussed later, which reveal two distinct glass transition temperatures for the mixtures studied. Similar behavior was observed by Grzybowska et al. in a mixture of ibuprofen and acetylated maltose.^18^

### Dielectric Studies of Neat Materials

First, we measured dielectric spectra of neat OTP in the supercooled liquid state over the temperature range 243 to 277 K (see Figure 3a). The structural relaxation process (α-relaxation), associated with whole-molecule motions, shifts to higher frequencies as temperature increases. At 275 K, its amplitude gradually decreases, which is attributed to crystallization and the resulting immobilization of molecules within the crystal lattice. The differences in the crystallization temperatures determined by DSC and BDS (*T*_c_^DSC^ = 290 K and *T*_c_^BDS^ = 275 K) are most likely due to differences in heating rate. It is worth mentioning that some authors were able to measure dielectric spectra in a broad temperature range without recrystallization of the supercooled OTP.^19^

**Figure 3:**
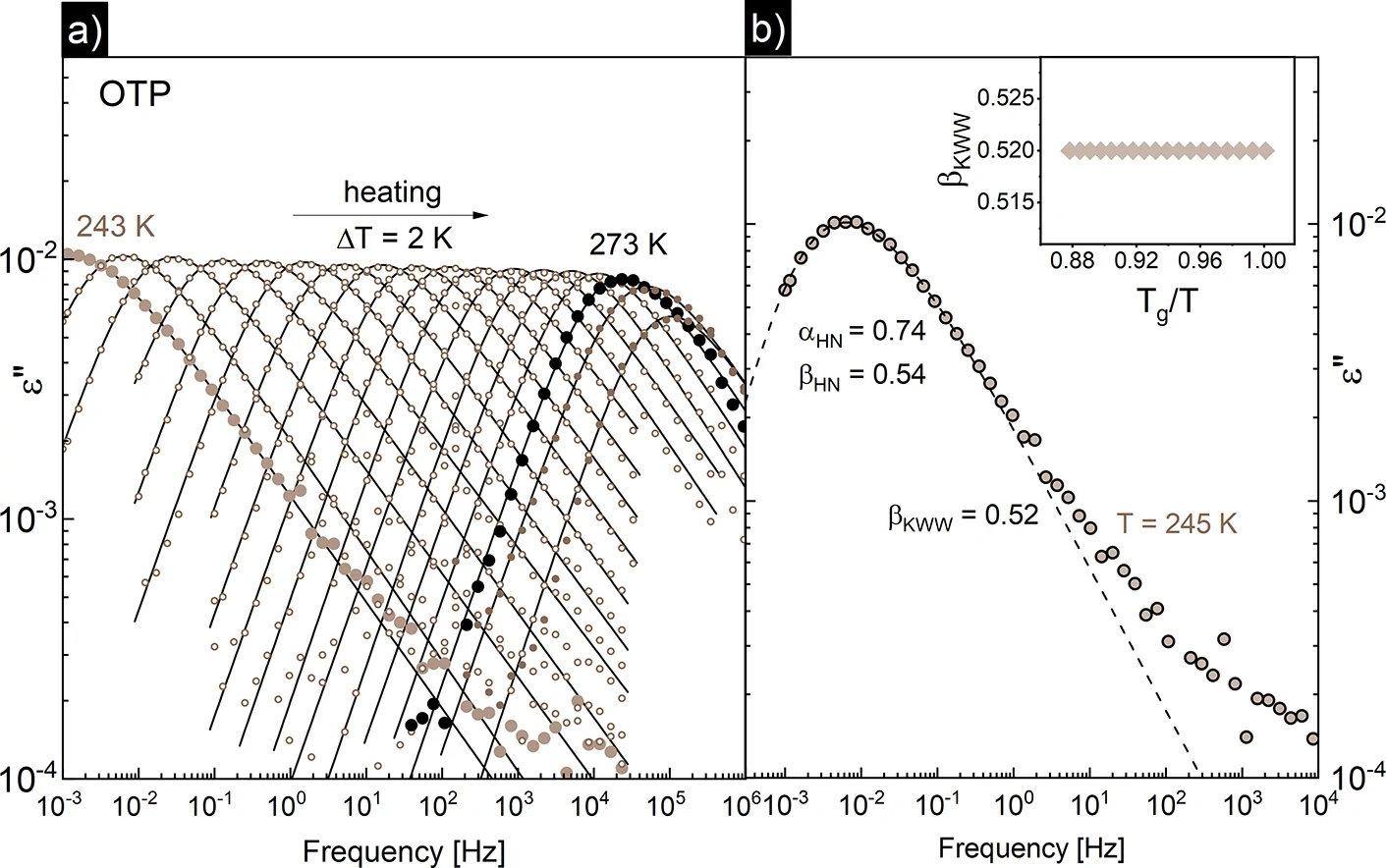
a) Dielectric response of *ortho*-terphenyl (OTP) over the tested temperature range. Full gray small points indicates partially crystallized spectra. Solid lines indicates HN fit functions. b) Selected spectrum (*T* = 245 K) near the glass transition (circles) with the KWW fit function (dashed line). The inset contains the temperature evolution of the β_KWW_ for OTP.

To obtain a more comprehensive characterization of the relaxation behavior of the investigated compound, the data shown in Figure 3a were analyzed using the Havriliak–Negami (HN) function with the additional term added describing the dc-conductivity contribution:

1 $$\varepsilon^{*}=\frac{\sigma }{\varepsilon_{0}\omega }+\varepsilon_{\infty }+\frac{\Delta \varepsilon }{{\left(1+{\left(i\omega \tau_{HN}\right)}^{\alpha }\right)}^{\beta }}$$

where σ/ε_0_ω defines the dc-conductivity contribution, ε_∞_ is the high-frequency permittivity limit, Δε is the dielectric strength, ω is the angular frequency, τ_HN_ corresponds to the Havriliak–Negami relaxation time, and the exponents α and β are the shape parameters.^20^ The dielectric spectra of the OTP in the supercooled liquid state were fitted satisfactorily with a single HN function. Structural relaxation times, τ_α_^OTP^, were then determined using the following equation:

2 $$\tau_{\alpha }=\tau_{HN}{[sin\left(\frac{\pi \alpha }{2+2\beta }\right)]}^{-1/\alpha }{[sin\left(\frac{\pi \alpha \beta }{2+2\beta }\right)]}^{1/\alpha }$$

The temperature evolution of τ_α_^OTP^ in the supercooled region (presented in Figure 7a) exhibits a non-Arrhenius behavior; thus, we employed the Vogel–Fulcher–Tammann (VFT) function to describe this dependence:

3 $$\tau_{\alpha }=\tau_{0}\exp\left(\frac{DT_{0}}{T-T_{0}}\right)$$

where τ_0_ is the pre-exponential factor, D determines the deviation from Arrhenius behavior, and T_0_ is the so-called ideal glass temperature.^21–23^ The resulting fit parameters are summarized in Table 1. Since an α-relaxation time of 100 s (log τ_α_ = 2) is conventionally taken to define the glass transition temperature, VFT parameters were used to calculate *T*_g_^BDS^. The glass transition temperature determined using BDS for neat OTP is *T*_g_^BDS^ = 243 K, which agrees with literature reports but differs from the DSC result due to a different heating rate.^19^

**Table 1:** Calorimetric and Dielectric Parameters Characterizing the Relaxation Dynamics of the Investigated Materials^a^

	*T*_g_^DSC^ [K]	*T*_g_^BDS^ [K]	log τ_0_ [s]	D	T_0_ [K]	β_KWW_ ∼ T_g_
OTP	249	243	–20.1 ± 0.3	17.6 ± 0.8	181.0 ± 1.3	0.52
*para*-F/OTP	253	252^d^	–18.8 ± 0.3^b^	20.7 ± 0.8^b^	176.3 ± 1.4^b^	0.79
			–11.3 ± 0.1^c^	3.7 ± 0.1^c^	232.3 ± 0.7^c^	
		246^e^	–17.1 ± 0.5	11.3 ± 0.9	196.6 ± 2.3	-
*ortho*-F/OTP	252	250^d^	–24.0 ± 1.5^b^	43.6 ± 8.5^b^	144.6 ± 8.2^b^	0.61
			–13.9 ± 3.7^c^	7.8 ± 7.8^c^	209.8 ± 33.3^c^	
		246^e^	–20.9 ± 0.4	19.3 ± 0.9	181.1 ± 1.6	-

a The VFT parameters were obtained exclusively from BDS data. For each binary mixture, the two parameter sets marked as VFT_lo‑T_ and VFT_hi‑T_ describe the slow, guest-related α-process, while the remaining set describes the fast, OTP-related α-process. Values of VFT fit parameters for neat *ortho*-F and *para-*F are available in ref 2.

b parameters for VFT_lo‑T_ fit.

c parameters for VFT_hi‑T_ fit.

d α_slow_.

e α_fast_.

To further characterize the α-relaxation, we analyze the width of the α-peak using the Kohlrausch–Williams–Watts (KWW) function:

4 $$\varphi \left(t\right)=\exp\left[-{\left(t/\tau_{KWW}\right)}^{\beta_{KWW}}\right]$$

in which β_KWW_ denotes the stretching parameter ranging from 0 to 1. For OTP, the β_KWW_ parameter is insensitive to temperature and remains 0.52 across the entire available temperature range. This result is in line with literature data obtained from dielectric measurements, which report values ranging from 0.5 to 0.52.^5,19,24,25^

Dielectric response studies of pure *ortho*-F and *para*-F were carried out in our previous paper.^2^ For *para*-F, a single, relatively narrow structural relaxation process is observed in the supercooled liquid state, with the corresponding β_KWW_ parameter decreasing from 0.90 at high temperatures to 0.64 as *T*_g_ is approached. The glass transition temperature determined from BDS is *T*_g_^BDS^ = 319 K, in good agreement with the DSC value (*T*_g_^DSC^ = 321 K).

In contrast, two relaxation processes were visible in the dielectric loss spectra of *ortho*-F. In addition to the dominant α-relaxation peak, a faster secondary relaxation (β-process) appears at higher frequencies, attributed to the internal rotation of the polar phenyl ring.^26–30^ This β-process is not observed for the *para*-F isomer because the dipole moment vector lies along the rotational axis, so dipole orientation does not change during internal rotation of fluorophenyl unit. The glass transition temperature determined from BDS data for *ortho*-F is *T*_g_^*BDS*^ = 310 K. The structural relaxation peak is broader than in *para*-F, with β_KWW_ = 0.5 over the available temperature range. The temperature dependence of corresponding structural relaxation times for both isomers, τ_α_^*ortho*‑F^ and τ_α_^*para*‑F^, obtained from HN fits are presented in Figure 7a, while the spectral shapes are compared in Figure 4.

**Figure 4:**
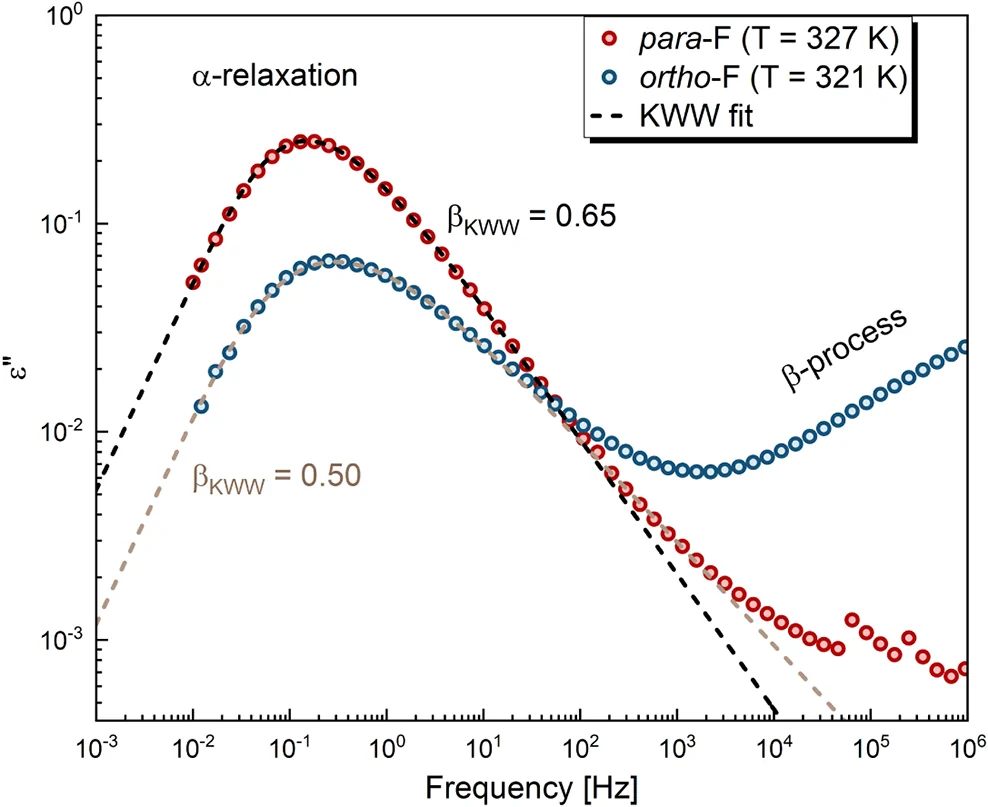
Comparison of dielectric loss spectra for neat *para*-F and *ortho*-F at selected temperatures.

### Dielectric Studies of Binary Mixtures

Next, we investigated the dielectric response of the *para*-F/OTP mixture (1:10 molar ratio) at various temperatures (see Figure 5). Analysis of the dielectric loss spectra, ε″(f), using the HN function revealed two distinct structural relaxation processes, denoted as $\alpha_{\mathrm{slow}}^{para‐F/\mathrm{OTP}}$ and $\alpha_{\mathrm{fast}}^{para‐F/\mathrm{OTP}}$ . Due to significant differences in the moments of inertia of the components, we assign a faster process to the motion of OTP molecules, whereas the slower one to the reorientation of *para*-F molecules.

**Figure 5:**
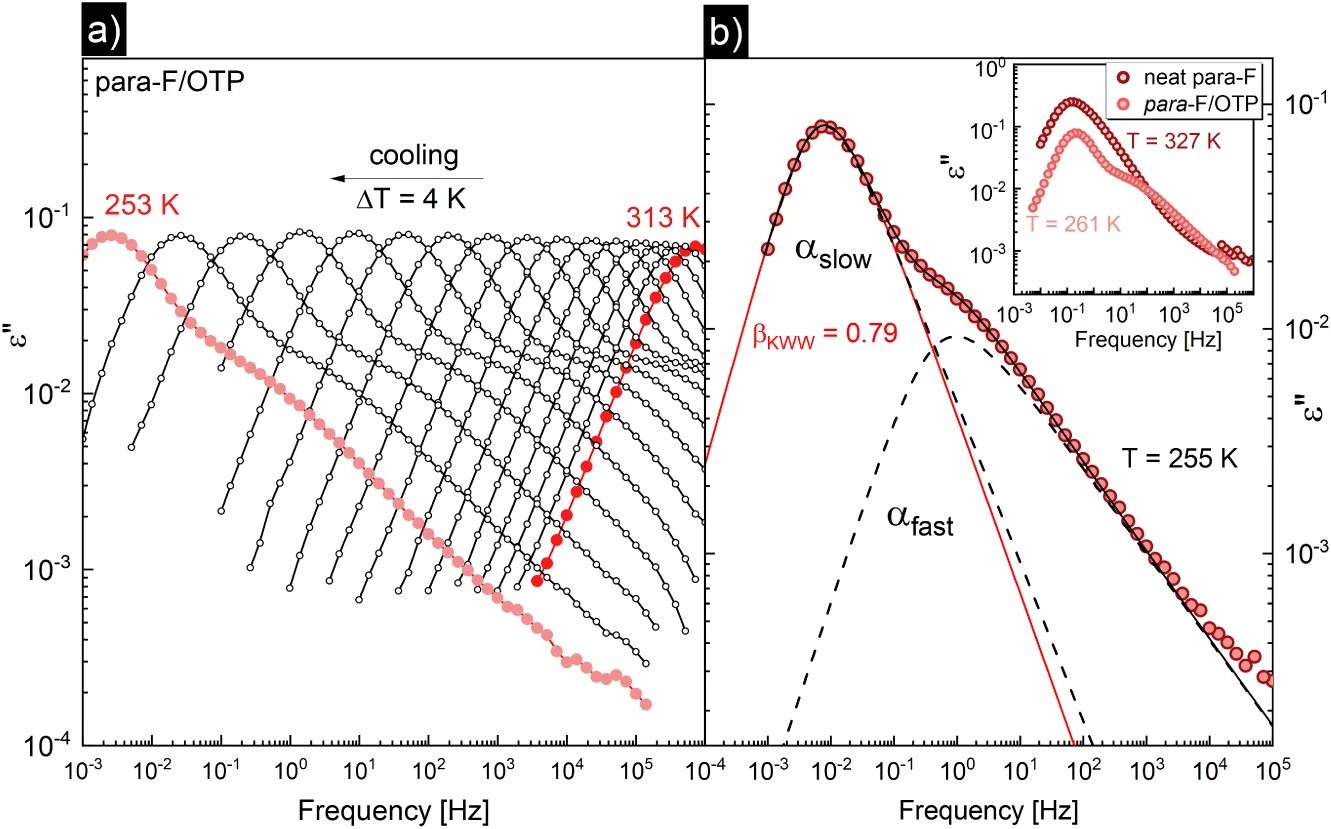
a) Dielectric response of *para*-F/OTP mixture over the tested temperature range. b) Selected spectrum near the glass transition with the HN fit function (solid black line), contributing functions (black dashed lines), and β_KWW_ fit function (red solid line). Inset shows the comparison of selected spectra at the same relaxation time for neat *para*-F and the *para*-F/OTP mixture.

The corresponding characteristic relaxation times of both processes, ${\tau \alpha }_{\mathrm{slow}}^{para‐F/\mathrm{OTP}}$ and ${\tau \alpha }_{\mathrm{fast}}^{para‐F/\mathrm{OTP}}$ , determined from HN fits are shown in Figure 7d. Stickel’s differential analysis indicated that their temperature dependence cannot be a single VFT function. In particular, this method enables verification of VFT fitting and facilitates identification of possible dynamic crossover temperatures.^31^ Consequently, two VFT functions are required for ${\tau \alpha }_{\mathrm{slow}}^{para‐F/\mathrm{OTP}}$ with crossover in 288 K (Figure 7e).

Interestingly, the VFT curves for the $\alpha_{\mathrm{slow}}^{para‐F/\mathrm{OTP}}$ and $\alpha_{\mathrm{fast}}^{para‐F/\mathrm{OTP}}$ processes do not merge near log τ_α_ = 2, indicating the presence of two glass transitions: *T*_g_^BDS^ = 252 K and *T*_g_^BDS^ = 246 K for the slow and fast processes, respectively. As discussed in the thermal analysis section, such a small difference in *T*_g_ may remain unresolved in DSC. However, analysis of dielectric data clearly separates the two glass transitions in the *para*-F/OTP mixture.

The spectral shape of the α_slow_ process was analyzed using the KWW function. The β_KWW_ parameter ranges from 0.9 at high temperatures to 0.79 near *T*_g_, indicating that the α_slow_ process is significantly narrower near the glass transition than neat *para*-F (see inset of Figure 5b). A detailed discussion of this behavior is presented later.

We further investigated the dielectric response of the *ortho*-F/OTP mixture (1:10 molar ratio) in the supercooled liquid state (see Figure 6). In this case, the dielectric spectra are more complex than those of the *para*-F/OTP mixture. Analysis of the ε″(f) data using the HN function reveals that the structural relaxation comprises two overlapping α-relaxation components, $\alpha_{\mathrm{slow}}^{ortho‐F/\mathrm{OTP}}$ and $\alpha_{\mathrm{fast}}^{ortho‐F/\mathrm{OTP}}$ , as well as an additional secondary β-relaxation at higher frequencies. As in the pure *ortho*-F, the β-process originates from internal rotation of the phenyl ring. The bimodal α-relaxation arises from the differing time scales of molecular motion for *ortho*-F $\left(\alpha_{\mathrm{slow}}^{ortho‐F/\mathrm{OTP}}\right)$ and OTP $\left(\alpha_{\mathrm{fast}}^{ortho‐F/\mathrm{OTP}}\right)$ in the mixture.

**Figure 6:**
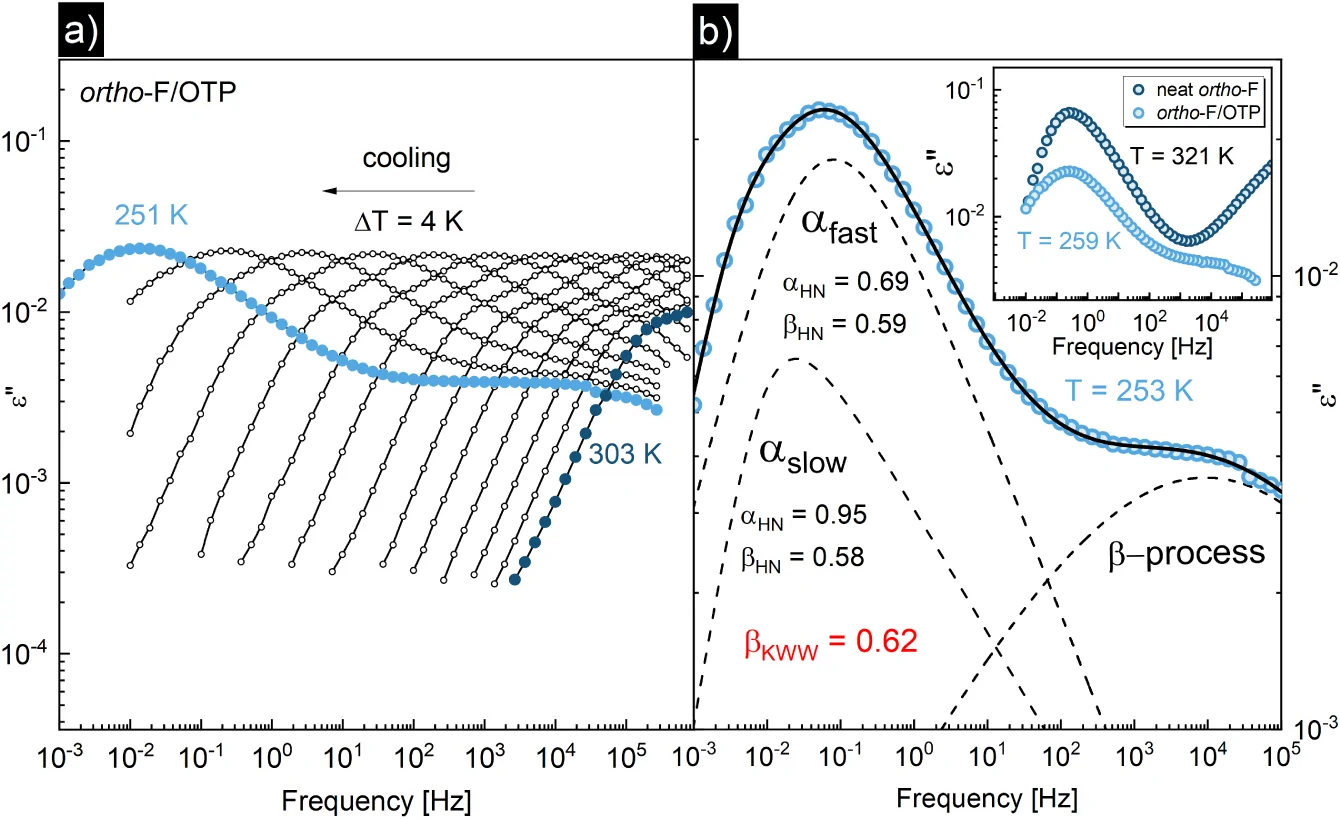
a) Dielectric response of *ortho*-F/OTP mixture in supercooled liquid state. b) Selected spectrum near the glass transition with the HN fit function (solid black line) and contributing functions (black dashed lines), and β_KWW_ fit function (red solid line). The inset shows a comparison of selected spectra at the same relaxation time for neat *ortho*-F and the *ortho*-F/OTP mixture.

As in neat *ortho*-F, the fitting of the temperature dependence of relaxation times for $\alpha_{\mathrm{slow}}^{ortho‐F/\mathrm{OTP}}$ process using a single VFT function are insufficient, as evidenced by the Stickel analysis (Figure 7e). Thus, two VFT functions were applied with crossover in 285 K. Again, the structural relaxation times of the slow and fast components do not merge at log τ_α_ = 2, indicating the presence of two glass transitions. The corresponding values are *T*_g_^BDS^ = 250 K and *T*_g_^BDS^ = 246 K for $\alpha_{\mathrm{slow}}^{ortho‐F/\mathrm{OTP}}$ and $\alpha_{\mathrm{fast}}^{ortho‐F/\mathrm{OTP}}$ , respectively.

**Figure 7:**
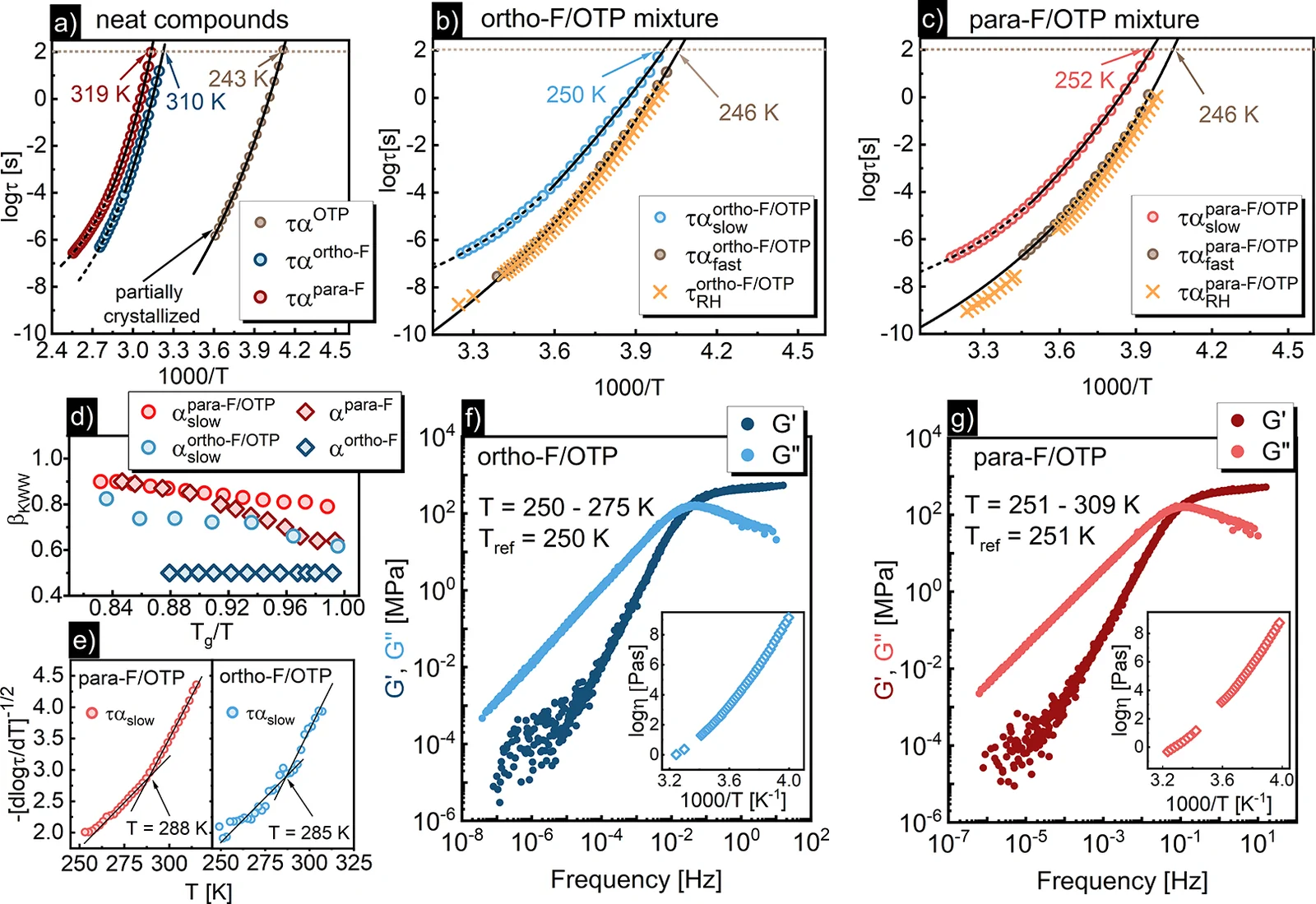
a) Relaxation map for neat *para*-F, *ortho*-F and OTP. b) and c) are relaxation map of *ortho*-F/OTP and *para*-F/OTP mixtures, respectively. The solid line represents VFT_lo‑T_, while the dashed line is VFT_hi‑T_. The arrows indicate the glass transition temperatures for relaxation time log τ_α_ = 2. d) Temperature dependence of the β_KWW_ value for neat *para*-F and *ortho*-F and for the α_slow_ process in *para*-F/OTP and *ortho*-F/OTP mixtures. e) Stickel analysis of α_slow_ process in *para*-F/OTP and *ortho*-F/OTP mixtures. Master curves of storage, G′, and loss, G″, modulus of f) *ortho*-F/OTP and g) *para*-F/OTP mixtures. Insets show the temperature dependence of viscosity in both mixtures.

Due to the strong overlap of the two structural α-relaxation processes in the investigated 1:10 mixtures, we performed additional BDS and DSC measurements for *ortho*-F/OTP mixtures with different compositions to examine whether changing the guest concentration could improve their spectral separation (see Figure 8). In particular, we aimed to determine whether increasing the *ortho*-F content would lead to a clearer distinction between the slow guest-related α-process and the fast OTP-related α-process. The additional measurements did not reveal a larger separation of the two relaxation time scales. Instead, the processes remained strongly overlapping and tended to approach each other with increasing guest concentration. At the same time, DSC measurements reveal only a single glass transition step for all investigated mixtures. Its position shifts systematically from *T*_g_ = 252 K for the 1:10 *ortho*-F/OTP mixture to 255 K for 1:5, 275 K for 1:1, and 298 K for 5:1. Thus, increasing the guest concentration does not result in the appearance of two calorimetrically distinguishable glass transitions.

**Figure 8:**
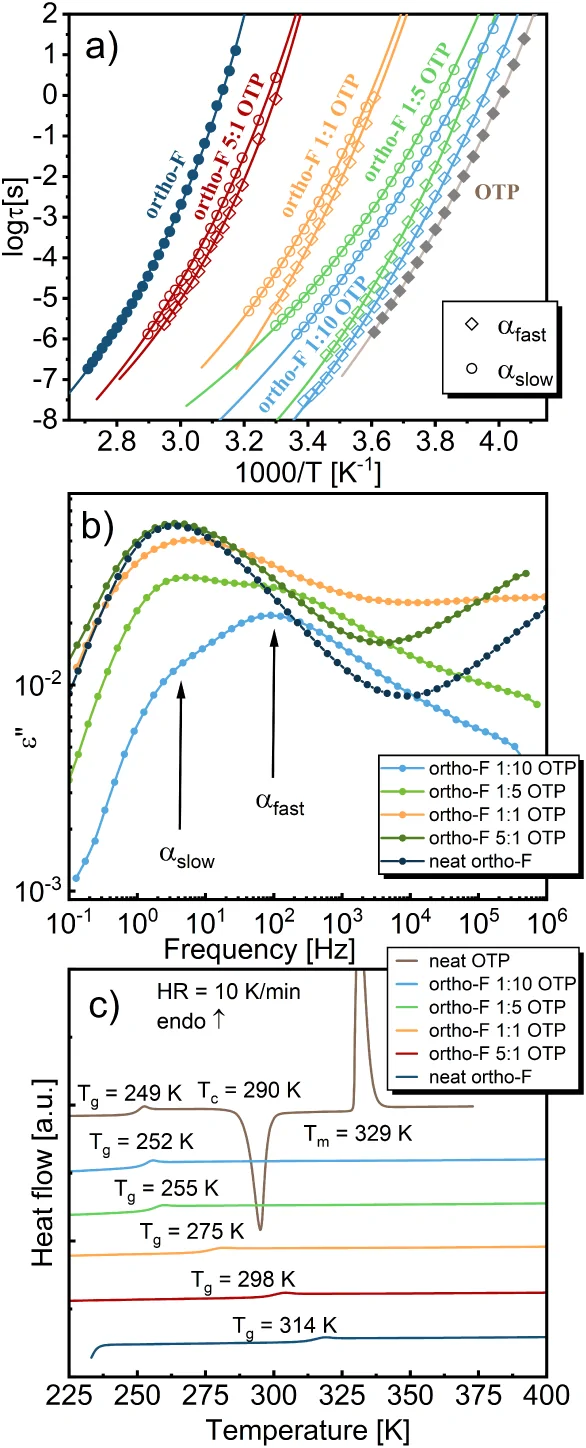
Concentration dependence of the dynamics of *ortho*-F/OTP mixtures: (a) relaxation map showing the guest-related (α_slow_) and OTP-related (α_fast_) relaxation times for different compositions; (b) comparison of representative dielectric loss spectra at comparable α_slow_ relaxation times; and (c) DSC thermograms of *ortho*-F/OTP mixtures with different compositions.

Because the α-relaxation components in the *ortho*-F/OTP mixture strongly overlap, a direct determination of the KWW parameters is subject to considerable uncertainty. Therefore, the β_KWW_ parameter was estimated from the HN shape parameters using an empirical relation: *β*_*KWW*_ ≈ (*α^HN^* *β* ^*HN*^)^1/1.23^.^32^ The resulting β_KWW_ values decrease from 0.73 at high temperatures to 0.62 near *T*_g_. Compared to the α-relaxation in neat *ortho*-F, the spectral shape of $\alpha_{\mathrm{slow}}^{ortho‐F/\mathrm{OTP}}$ process becomes both narrower and more temperature dependent.

The presented results reveal a clear, somewhat unexpected effect. Upon dilution in a weakly polar OTP matrix, the dielectric relaxation associated with the reorientation of the large, anisotropic molecules (*para*-F and *ortho*-F) becomes significantly narrower than in neat systems. This is reflected in a higher β_KWW_ stretching parameter, indicating reduced dynamic heterogeneity. Such behavior contrasts with the scenario commonly reported in the literature, where mixing components of different polarity and size typically increases dynamic heterogeneity and broadens the relaxation spectrum.^14–17,33–35^

The observed narrowing of the α-relaxation peak can be understood as a consequence of dynamic homogenization of the guest molecules within the OTP matrix. This homogenization originates from two cooperative factors: (i) the significantly larger size of the guest molecules relative to the surrounding OTP molecules, which enables spatial averaging of local dynamic heterogeneities and this interpretation is consistent with the work of Cicerone et al., who demonstrated that the relaxation function becomes progressively narrower as the size of the probe molecule increases relative to the surrounding OTP molecules,^36^ and (ii) the reduction of direct intermolecular guest–guest interactions, due to dilution in the weakly polar OTP host. Together, these effects reduce the distribution of local relaxation environments and consequently lead to a narrower α-relaxation spectrum.

### Rheological Studies of Binary Mixtures

The next step in our study involves rheological experiments to determine mechanical relaxation times (τ_RH_). The measured storage, G′(f), and loss, G″(f), moduli, as well as viscosity, η­(T), are presented in Figure 7f,g. Based on viscosity values and the high-frequency shear modulus *G*_∞_ determined from G′(f) spectra, the relaxation times were calculated using Maxwell’s equation *τ_RH_* = *η/G*_∞_.^37^ The extracted *G*_∞_ value for the *para*-F/OTP mixture is *G*_∞_ = 528 MPa, while for the *ortho*-F/OTP mixture it is *G*_∞_ = 539 MPa.

The resulting τ_RH_ relaxation times are shown in Figure 7b,c, together with the τ_α_ values determined from BDS for the slow and fast components of the α-process. In both mixtures, the τ_RH_ relaxation times almost perfectly coincide with the τα_fast_ times previously assigned to the α-relaxation of the OTP matrix. This agreement indicates that, in the investigated mixtures, the reorientation dynamics of OTP are coupled to viscosity throughout the supercooled regime. As OTP is the dominant component, it governs the viscous response and thereby determines the mechanical relaxation behavior. Such coupling is consistent with the literature reports, as Sillescu et al. demonstrated that neat OTP does not exhibit the violation of the DSE rule between rotational correlation time τ_c_ and viscosity.^38,39^

Now we compare the τα_slow_ and τ_RH_ in both mixtures. Qualitatively, a clear difference is observed in their temperature evolution depending on the fluorophenylene isomer. In the *para*-F/OTP mixture, τα_slow_ and τ_RH_ exhibit nearly parallel temperature dependences over most of the investigated range, with only minor deviations appearing at higher temperatures. In contrast, the *ortho*-F/OTP mixture shows a gradual convergence of τα_slow_ and τ_RH_ as the temperature approaches the glass transition temperature, suggesting an increasing degree of coupling between the probe dynamics and the matrix-controlled viscosity.

To quantify this behavior, the validity of the DSE relation was evaluated by comparing τα_slow_ and τ_RH_. In its classical form, the DSE relation predicts a linear proportionality between these two time scales. Deviations from this behavior can be described within the fractional Debye–Stokes–Einstein (FDSE) framework, expressed as τα_slow_ ∝ τ_RH_^–ξ^, where the exponent ξ quantifies the degree of coupling. A value of ξ = 1 corresponds to DSE behavior, whereas ξ < 1 indicates partial decoupling of the probe’s orientational relaxation from the viscosity-controlled structural dynamics of the host matrix.^40–42^ To determine ξ, we constructed a double-logarithmic plot of log­(τα_slow_) versus log­(τ_RH_) (see Figure 9a). For *para*-F/OTP, the slope yields ξ = 1.00, indicating a proportional change of the relaxation time with viscosity. In contrast, for *ortho*-F/OTP, the slope is ξ = 0.95 and this indicates a consistent speed-up of τα_slow_ relative to τ_RH_ across the whole range, and thus a systematic decoupling between the probe relaxation time and the viscosity of the host matrix.

**Figure 9:**
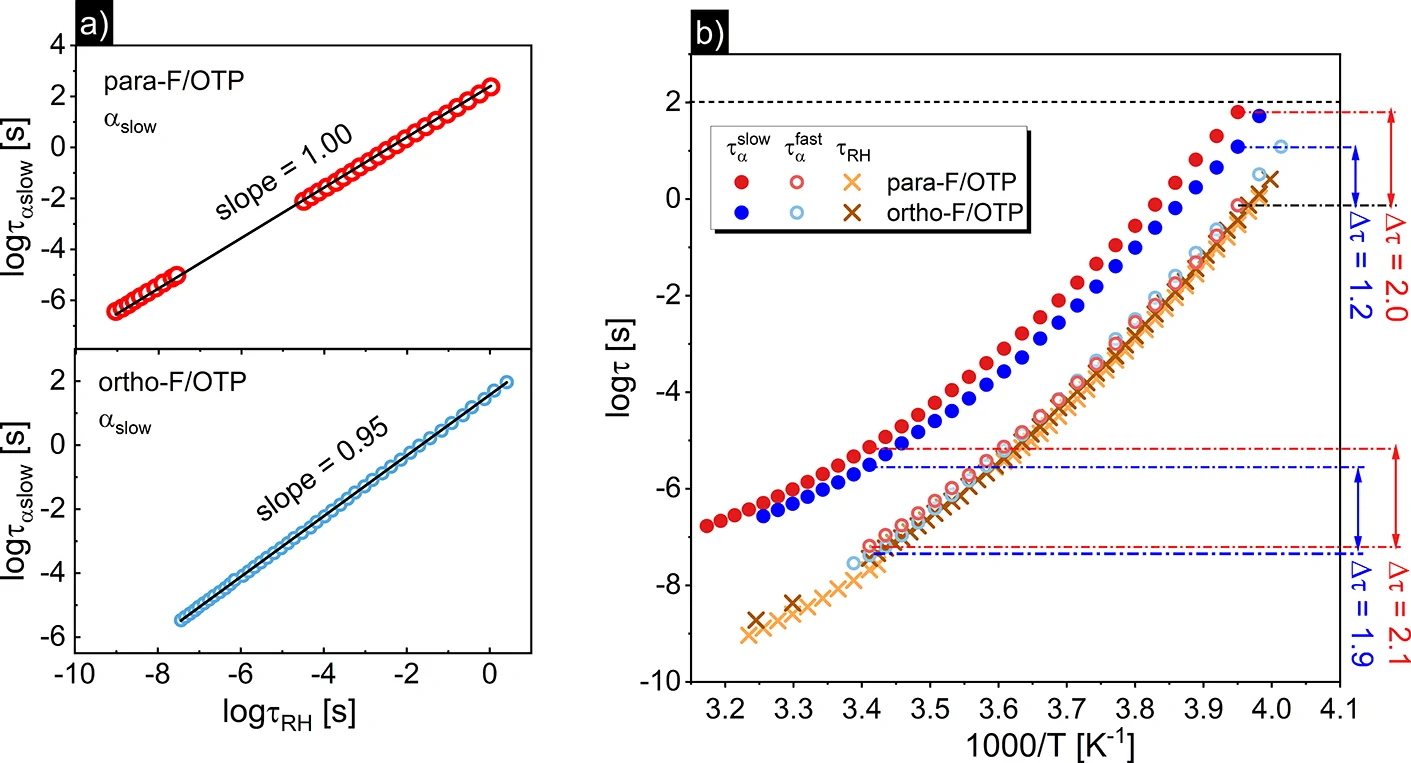
a) Double logarithmic plot comparing dielectric and mechanical relaxation times. b) Relaxation map comparing the structural and mechanical relaxation times of all the investigated mixtures. Dot-dashed lines in the respective colors indicate the distance between $\tau_{\alpha }^{\mathrm{slow}}$ and $\tau_{\alpha }^{\mathrm{fast}}$ at a given temperature.

Since τ_RH_ closely follows the α-relaxation time of the OTP matrix $\left(\tau_{\alpha }^{\mathrm{fast}}∼\tau_{RH}\right)$ the probe dynamics τα^slow^(T) can alternatively be analyzed in terms of their separation from the host-controlled structural relaxation τα^fast^(T) rather than in terms of viscosity-related τ_RH_. For this purpose, we introduce Δτ = log­(τα_slow_) – log­(τα_fast_). In this representation, DSE-like behavior corresponds to approximately constant Δτ over the investigated temperature range, whereas deviations from the DSE relation manifest as a systematic change in Δτ as T approaches *T*_g_. As shown in Figure 9b, Δτ for the *para*-F/OTP mixture changes only marginally (≈0.1 decades, i.e., within experimental uncertainty), confirming strong coupling to the host dynamics over the entire temperature range. In contrast, the *ortho*-F/OTP mixture exhibits a significantly larger variation of Δτ (≈0.7 decades), consistent with a pronounced and persistent decoupling of the probe relaxation from the matrix dynamics. Interestingly, this behavior is opposite to that observed by Raj et al. for neat systems. In that study, neat *ortho*-F did not show any breakdown of the DSE rule across the entire available temperature range. For neat *para*-F, relatively strong decoupling between relaxation times and viscosity was observed at temperatures slightly above *T*_g_. In neat systems, the validity of the DSE relation depends on which aspect of anisotropic molecular motion is being sampled: it breaks down when predominantly short-axis rotations are involved and remains valid when long-axis rotations dominate.

At this point, it is essential to emphasize the fundamental difference between the dynamics of pure liquids and the behavior of mixtures. In single-component systems, viscosity, structural relaxation, and dynamic heterogeneity are generated by the same molecules, and the observed degree of coupling or decoupling between relaxation times and viscosity reflects the material’s internal dynamics. The situation is different in probe-host mixtures, such as systems containing *ortho*-F or *para*-F dissolved in OTP. It is important to note that in probe-host mixtures, the mechanical relaxation time τ_RH_ primarily reflects the dynamics of the OTP matrix, which constitutes 90 mol % of the system. Consequently, the observed scaling exponents describe the coupling between the probe dynamics (τα_slow_) and the host-controlled structural relaxation.

Moreover, for anisotropic molecules, different aspects of molecular reorientation can be coupled to the matrix dynamics to various extents. Rotation around the short molecular axis, associated with a larger moment of inertia and a larger effective hydrodynamic volume, requires simultaneous reorganization of the surrounding matrix molecules on a relatively large spatial scale. In contrast, reorientation around the long molecular axis involves a smaller moment of inertia and a lower hydrodynamic volume. It can occur without the participation of a large number of OTP molecules.

From a molecular perspective, the measured differences in ξ can be related to the type of molecular motion probed by dielectric spectroscopy. For the *para*-F/OTP mixture, τα_slow_ predominantly reflects short-axes reorientations that are strongly coupled to the matrix dynamics, consistent with ξ ≈ 1. In the *ortho*-F/OTP mixture, the slightly reduced exponent (ξ ≈ 0.95) indicates that the probed relaxation involves motions that are marginally less constrained by the macroscopic viscosity, leading to a small but measurable deviation from linear DSE scaling.

## Conclusions

In this work, we investigated the relaxation dynamics of large, anisotropic fluorinated molecules embedded in a weakly polar OTP matrix, focusing on the interplay among anisotropic reorientation, the host’s structural dynamics, and macroscopic viscosity. Broadband dielectric spectroscopy, calorimetry, and shear rheology enabled us to disentangle the dynamical responses of the probe molecules and the host matrix and to assess the validity of the DSE relation in the investigated probe-host systems.

All investigated mixtures exhibit two distinct α-relaxation processes: a fast process associated with the structural relaxation of OTP and a slower process originating from the orientational relaxation of the sizable molecules. While calorimetric measurements reveal only a single glass transition dominated by OTP, dielectric spectroscopy clearly resolves the spectral signatures of mixture components. Notably, dilution of the sizable molecules in OTP leads to a pronounced narrowing of their dielectric α-relaxation spectra, reflected in increased β_KWW_ values. This behavior is a consequence of dynamic homogenization of the guest molecules within the OTP matrix.

Rheological measurements show that the mechanical relaxation time derived from viscosity coincides with the fast dielectric α-process of OTP, demonstrating that the viscosity of the mixtures is governed exclusively by the dynamics of the host. A comparison between the probe relaxation time and the mechanical relaxation time reveals distinct DSE behavior for different isomers. The *para*-F/OTP mixture exhibits ideal DSE coupling, whereas *ortho*-F/OTP shows systematic decoupling over the entire temperature range. This inversion relative to the behavior of neat systems highlights that DSE compliance in mixtures is controlled by the character of the dielectrically probed reorientation mode and the degree of its coupling to the surrounding matrix.
